# Exploring Muslim Women’s Reproductive Health Needs and Preferences in the Emergency Department

**DOI:** 10.5811/westjem.58942

**Published:** 2023-08-22

**Authors:** Anum Naseem, Morgan Majed, Samantha Abdallah, Mayssa Saleh, Meghan Lirhoff, Ahmad Bazzi, Martina T. Caldwell

**Affiliations:** * Wayne State University School of Medicine, Department of Emergency Medicine, Detroit, Michigan; † Henry Ford Hospital Department of Emergency Medicine, Detroit, Michigan

## Abstract

**Objective:**

We explored individual Muslim women’s reproductive healthcare experiences, preferences, beliefs, and behaviors in the emergency department (ED) and in general.

**Methods:**

This was a qualitative study conducted at a community ED using semi-structured interviews with a piloted interview guide. We interviewed participants awaiting care in the ED with the following criteria: female gender; English or Arabic speaking; aged ≥18 years; and self-identified as Muslim. We conducted interviews in both English and Arabic until thematic saturation was reached. Transcripts were coded using an iteratively developed codebook, maintaining intercoder agreement greater than 80%. We used an inductive thematic analysis to identify themes, and results were interpreted in the context of interview language and patient’s age.

**Results:**

We interviewed 26 Muslim-identified female ED patients. We found that cultural representation and sensitivity among ED staff mitigated discrimination and promoted inclusion for Muslim ED patients. However, assumptions about Muslim identity also impacted the participants’ healthcare. Most participants endorsed a preference for a female clinician for their reproductive healthcare in general, but not necessarily for other areas of medicine. Clinician cultural concordance was not always preferred for participants in the ED due to fears about the loss of confidentiality. Marital status impacted beliefs about reproductive and sexual health in the context of Muslim identity. Overall, family planning was acceptable and encouraged in this patient population.

**Conclusion:**

The themes elucidated in this study may guide clinicians in developing culturally sensitive practices when providing reproductive healthcare to the Muslim population.

Population Health Research Capsule What do we already know about this issue?

*Best practice care models for Muslim patients that have been published to improve cultural competency among clinicians are not derived from patient preferences.*

What was the research question?

*We aimed to explore Muslim women’s reproductive healthcare experiences and preferences in the ED and in general.*

What was the major finding of the study?

*Most women preferred a female clinician for their reproductive healthcare, but cultural concordance was not always desired due to fears about confidentiality. Family planning was acceptable, and desired in this patient population.*

How does this improve population health?

*The themes elucidated here may guide clinicians in developing culturally sensitive practices when providing reproductive healthcare to this population.*

## INTRODUCTION

Racial and ethnic minorities continue to have inequitable healthcare access and outcomes, from preventive measures to the treatment of acute illness.^1^^–^^4^ With an increasingly diverse patient population, much focus has been on improving patient-clinician cross-cultural interactions to prevent stereotyping, biases, and lack of trust that impede the delivery of quality care.^2^ Models of cultural competence emphasize a patient-centered approach to enhance healthcare delivery to minority groups with cultural and linguistic differences.^5^ This is especially important in the emergency department (ED), a safety-net care setting in which clinicians do not typically have established relationships with patients.^6^^,^^7^

Health outcomes and cultural preferences of the Muslim population are not well established in the healthcare literature or practice guidelines.^8^ An estimated 3.3 million Muslims live in the United States today, making Islam the third most prominent religion in the country.^9^ The Muslim population is growing and projected to reach 8.1 million by 2050, largely due to increased immigration.^10^ Considering the prevalence of Muslims nationally, it is important that clinicians are well trained in delivering care that is sensitive to the unique perspectives and beliefs of these patients.

Ensuring that clinicians are knowledgeable about Muslim patients’ preferences regarding reproductive health is especially important in the ED where patients often present for obstetric or gynecological complaints and there is no established patient-clinician relationship.^11^ Although best practices for care of Muslim patients in the ED have been published, there is a lack of literature that is derived from patient preferences that specifically focuses on reproductive healthcare for female Muslims in the ED.^11^^–^^14^ Moreover, the Muslim population constitutes a very heterogenous population that includes many different ethnic groups, and in which religious identity is often confounded by specific cultural values. Understanding perspectives of individual patients can help elucidate how to best address the diversity of identity within this population.^15^ Therefore, we aimed to explore Muslim women’s reproductive healthcare experiences, preferences, beliefs, and behaviors in the ED and in general.

## METHODS

### Study Design and Setting

We conducted semi-structured, face-to-face interviews using a piloted interview guide with female Muslim patients of reproductive age presenting to a community ED located in a midwestern suburb with a large Muslim population. The study was approved by the institutional review board at the institution where data collection occurred. To ensure our interview guide was culturally sensitive, we performed cognitive interviews in English while piloting the interview guide.

Two team members (AN and MS) conducted all study activities in English or Arabic according to the participant’s preference. AN, a third-year medical student, conducted all of the English interviews, and MS worked as a registered nurse at the study site and conducted all of the Arabic interviews. Both AN and MS are reproductive-aged, cisgender females who identify as Muslim, but neither wear the hijab. AN is South Asian and is familiar with the community through her medical education activities and volunteerism. MS is Lebanese and a life-long member of the local Muslim community. Both were new to qualitative research and were trained by MC, who has extensive training and experience in qualitative methods and community-based participatory research. Note that all Arabic research documents were professionally transcribed and certified by the health system’s approved company, as well as reviewed by multiple Arabic-speaking members of the study team.

### Selection of Participants

While patients awaited medical treatment in the ED, a study team member screened the electronic health record (EHR) track board for inclusion in this study and approached potentially eligible participants. Participants who met the following criteria were included: 1) female gender per the EHR; 2) age ≥18 years per the EHR; and (3) self-identified as Muslim. Any individual who was identified by the EHR to meet the first two inclusion criteria were approached and asked whether they self-identified as Muslim to avoid profiling. Individuals who met the following criteria were excluded: 1) in physical, mental, or emotional distress, including Emergency Severity Index 1 (the highest acuity); 2) prisoners; 3) cognitively delayed; or 4) could not understand and converse about reproductive health in English or Arabic. Interviews were conducted in the ED patient rooms without the presence of family members in the majority of cases.

### Measurements

Participants completed a brief screening questionnaire to obtain demographic information. Written informed consent was obtained from all participants in their preferred language. Participants received a $20 cash compensation for participating in the 30- to 40-minute interviews. Interviews were audio recorded, transcribed, and de-identified by a professional transcription company. Arabic transcripts were subsequently translated into English and verified by MH and AB, who are Arabic-speaking, Muslim resident physicians. Interviewers documented field notes of their observations and experiences for each interview, which were used to contextualize interview data during analysis.

### Analysis

Interviews were collected until thematic saturation was reached or when recurring themes were identified in the manuscripts, which was found to be at 26 interviews. No repeat interviews needed to be conducted. The study team developed a codebook using axial and open coding, resolving disagreements using a consensus process. The codebook was iteratively revised throughout coding to include emerging content. Prior to coding, and every five transcripts thereafter, coders performed an intercoder agreement trial and maintained intercoder agreement at or above 80%. Using qualitative data analysis software Dedoose version 7.0.23 (SocioCultural Research Consultants, LLC, Los Angeles, CA), two trained coders (MM, a Muslim graduate student and SA, a Muslim medical student) employed codes from the codebook to the transcribed and de-identified interviews. AN used an inductive thematic process to analyze the text data. Findings were validated by the entire research team, which is comprised of mostly Muslim members and two non-Muslims. Thematic results were converged with demographic data obtained in the questionnaire.

## RESULTS

### Characteristics of Study Subjects

We interviewed 26 participants, of whom 14 were English-speaking and 12 were Arabic-speaking (Table 1). The majority of participants (16) were of reproductive age, defined as 18–50 years old; 19 were US citizens; 23 had publicly funded health insurance; and 14 identified as having more traditional Islamic religious views. The Arabic-speaking group was older than the English-speaking interviewees (39 vs 33 years old). All the Arabic-speaking participants were born outside the US, and this group had lower educational levels with 41.6% reporting an eighth-grade education or less. The English-speaking population mostly identified as Lebanese (11), whereas the ethnicity most often represented among the Arabic-speaking group was Yemeni (six participants).

**Table 1.** Participant demographics of English- and Arabic-speaking interviews conducted in the emergency department.^*^

	English (n = 14)	Arabic (n = 12)	Total (N = 26)
Average age, years	33	39	38
ED visits in the last 12 months, mean	1.6	2.9	2.7
Place of birth (%)			
In the United States	42.8 (6)	0 (0)	23.0 (6)
Outside the United States	57.2 (8)	83.3 (10)	69.2 (18)
Citizenship (%)			
US citizen, by birth	50 (7)	0 (0)	26.9 (7)
US citizen, by naturalization	28.5 (4)	50 (6)	38.4 (10)
US citizen, born abroad by parents who are US citizen	14.2 (2)	0 (0)	7.6 (2)
Not a U.S. citizen	7.1 (1)	41.6 (5)	23 (6)
Health insurance (%)			
Public insurance	92.8 (13)	83.3 (10)	88.4 (23)
Private insurance	7.1 (1)	16.6 (2)	11.5 (3)
Ethnicity (%)			
Iraqi	14.2 (2)	25 (3)	19.2 (5)
Lebanese	78.5 (11)	16.6 (2)	50 (13)
Palestinian	0 (0)	8.3 (1)	3.8 (1)
Yemeni	7.1 (1)	50 (7)	26.9 (8)
Marital status (%)			
Never married	28.6 (4)	8.3 (1)	19.2 (5)
Married	50 (7)	66.7 (8)	57.6 (15)
Divorced	14.3 (2)	8.3 (1)	11.5 (3)
Separated	0 (0)	8.3 (1)	3.8 (1)
Widowed	7.1 (1)	0 (0)	3.8 (1)
Religiosity (%)			
Traditional	64.3 (9)	41.6 (5)	53.8 (14)
Neither traditional nor non-traditional	21.4 (3)	25 (3)	23 (6)
Non-traditional	7.1 (1)	16.6 (2)	11.5 (3)
Highest grade/degree completed (%)			
8^th^ grade or less	7.1 (1)	41.6 (5)	23 (6)
High school graduate/GED	50 (7)	8.3 (1)	30.8 (8)
Some college or associate’s degree	28.6 (4)	33.3 (4)	30.8 (8)
Bachelor’s degree or higher	14.3 (2)	8.3 (1)	11.5 (3)
Difficulty for you or your household to pay bills in last 12 months, (%)			
Hard	50 (7)	33.3 (4)	42.3 (11)
Neither hard nor easy	35.7 (5)	0 (0)	19.2 (5)
Easy	7.1 (1)	50 (6)	26.9 (7)

*ED*, emergency department; *GED*, General Educational Development.

* Not all data add up to 100% because of missing data or rounding.

### Main Results

We grouped themes into two main categories: 1) impact of Muslim identity on reproductive health experiences in general; and 2) impact of Muslim identity on reproductive health preferences, beliefs, and behaviors (Table 2). The supporting quotes are formatted as follows: interview language (E = English, A = Arabic); participant number; and participant age in years (y).

**Table 2.** Supporting quotes from study participants.

Themes	Illustrative quotes
I. Cultural representation and cultural sensitivity mitigate experiences of discrimination and promote feelings of inclusion	• “They’re very understanding, especially around here in the community” (E13, 34y). • “I feel like if I were to go out of state or anywhere, everybody would look at me differently, just because I’m a Muslim” (E9, 20y). • “Where we go we see racism so we don’t feel comfortable. Everywhere, not only in the hospital. Thank God, I wasn’t treated with racism in the hospital. They treat everyone equally” (A1, 29y). • “I have been here for a very long time, [doctors] never differentiate [between a non-Muslim and a Muslim]” (A12, 49y). • “[The doctors] are nice and they have mercy, it doesn’t make a difference for them if we are Muslims or not…they have more mercy than we do” (A4, 60y). • “I feel that [the doctors] are good…they deal in a nice way, they don’t discriminate whether you are Sunni, Shia, American or Arab” (A7, 27y). • “We all get treated the same [in the ED]” (E3, 33y). • “When I came [to the ED], they treated me in a very nice way, in gynecology, they respect that I wear a Hijab and they take care of me” (A12, 49y). • “They’re very understanding [of my Hijab] especially around here in the community. Most of the doctors know and they’re aware of all that, so they do whatever they can to accommodate it (E13, 34y).
II. Assumptions about Muslim identity	• “When you walk in and you’re covered…a male doctor will walk in, they do get a little ‘Are you okay with [me being here]?’ or ‘Do you want us to, you know--?’…but not if it wasn’t a covered woman or a Muslim woman” (E2, 31y). • “Just because they see you covered up doesn’t mean you don’t speak their language. I think they have that mentality, but it’s far from the truth” (E8, 27y). • “If they see I don’t have a wedding ring on and I’m Muslim, they’re like ‘Oh, so you’re not pregnant for sure…she’s a virgin. She doesn’t drink. I’m going to quickly go over this question’…whereas people can be varying levels of religiosity depending on whether they wear the headscarf or not” (E15, 24y).
III. Preference for a female clinician tends to be specific to reproductive health	• “Some problems should only be discussed with a female doctor…woman to woman there’s nothing to hide” (A5, 37y). • “If they have to do the vaginal test, yes [would prefer a woman]…because we are Muslims” (E11, 31y). • “Because he is a stranger, you know that in our religion that’s not acceptable, it is Haram unless if it is an emergency and her life is in danger, then it is acceptable….I am only talking about genital organs, but it is fine in other specialties.” (A8, 47y). • “I think [clinician gender] doesn’t matter except if I am going to have a gynecological exam” (A8, 47y). • “If it is a female doctor, I feel more comfortable because we are the same” (A2, 34y). • “When I gave birth to my eldest son, the female doctor wasn’t there, and I had to deal with a male doctor, it was embarrassing but I accepted it because it was an emergency case” (A1, 29y).
IV. Preference for non-Muslim/non-Arab clinician	• “I know that most of my race, subconsciously they will judge you regardless of if they tell you they don’t…I don’t like to be criticized or judged or looked at in a certain way just because of how I look or what I’m here for…what my blood results will come out to” (E2, 31y). • “They prefer to go to a Caucasian or any other race of psychologists than one just like them, in fear or worry that I will gossip in the community…and it works both ways. Like I would be scared too” (E15, 24y). • “I think there are a lot of girls that are younger. They get abortions. But they don’t have nobody to talk to …especially being Arab, you can’t. You have to go somewhere nobody knows about…The problem isn’t with the doctor. The problem is with the… whole environment. You really can’t say much in Dearborn” (E3, 33y). • “If I had done something or wanted to talk about it, I’ll be worried…if there’s people listening in the hallway, or if someone recognizes me” (E15, 24y).
V. Marital status impacts ideas about intercourse	• “In the past maybe like before I got married [preferred female doctor], before I had kids. But after all that it’s like, they’ve seen everything. You’re just open about it. You don’t care anymore” (E13, 34y). • “[Intercourse] is [not permissible] in our religion…only with her husband” (A8, 47y). • “Muslim girls don’t wear tampons if they’re not married yet” (E15, 24y). • “You’re not supposed to do [pelvic exams] until basically you’re married” (E9, 20y).
VI. Religious permissibility for contraception	• “I didn’t know that [birth control] had anything to do with religion” (E2, 31y). • “I would never take birth control nor would I want my daughters to…it’s not that [Islam is] against it. It’s not natural but then again, it is their choice but I would advise them don’t take it” (E10, 54y). • “[Birth control] is not something people talk about that much, but people our age, I think it’s not a big deal for us” (E15, 24y). • “It is normal to take birth control pills, if I don’t want to get pregnant, it is totally fine to take birth control pills” (A7, 27y). • “If the woman is weak and can’t support a new pregnancy because of several previous miscarriages, she should use birth control pills to protect her health or if the woman has enough children, she should use them as well” (A8, 47y). • “I’ve used contraceptive pills and that’s okay [in my religion]” (A11, 67y). • “I don’t know what Islamic religion says about birth control pills but personally I used to take them when I was in Lebanon. I didn’t think about religion at that time, but it is a bad thing to bring a baby to this world if you can’t take care of him” (A12, 49y). • “Religion is not against birth control pills…in fact it’s quite the opposite. It’s for these things, because you can’t risk getting pregnant every day” (A9, 57y).

*A*, Arabic-speaking participant; *E*, English-speaking participant; *ED*, emergency department; *y*, age in years.

#### Impact of Muslim Identity on Reproductive Health Experiences in General

*I. Cultural representation and cultural sensitivity mitigate experiences of discrimination and promote feelings of inclusion.* The most prevalent theme in the interviews addressed discrimination, cultural representation, and cultural sensitivity in healthcare in general. When interviewers asked participants whether they had ever felt discriminated against while receiving reproductive health services because of their religion, the majority of participants denied such experiences in the ED and in healthcare at large. For example, many participants seemed to agree that “I don’t see any difference [in receiving reproductive healthcare] whether I wear a hijab or not. They treat us the same way” (A2, 34y). Put more explicitly, “I’m treated the same by every doctor and nurse that I encounter, so I think [discrimination] is not a problem” (E15, 24y).

Many participants alluded to the unique cultural representation in the local community as being a major protective factor against discrimination and bias. One participant said, “coming in [to the ED] and seeing Muslim people…it just makes me more comfortable” (E15, 24y). Another participant described how “[in this hospital], since they know there’s a lot of Arabs and stuff, and they always have Arab nurses, they can communicate, so here it’s better” (E7, 35y) indicating that cultural representation is an important part of building comfort with clinicians. Additionally, some participants also suggested that, “a lot of the American doctors [in this community]…know our culture…but if you go outside like Florida and stuff…I heard that they would treat [Muslims] differently” (E7, 35y).

Notable exceptions to this theme seemed related to language and religious clothing, such as the hijab. One participant stated, “I’ve heard [a lot of stories of discrimination] and it usually [involved] women who didn’t speak English” (E8, 27y). Another participant concurred that “probably because [another Muslim woman] had an accent and [she] couldn’t speak English well, [the clinicians] were kind of rude to [her]” (E7, 35y). One participant shared a story of her support of another Muslim woman who wore a hijab, describing, “a little scarf lady…she couldn’t defend herself, so I had to defend her because [the clinicians were] talking like she’s stupid” (E6, 27y). One of the English-speaking participants explained that she had never encountered discrimination due to her Muslim religion, “maybe because I don’t wear a head scarf, it’s different. And I come out speaking English - you wouldn’t mistake me for a Muslim.” Despite her own experiences, she had heard stories of other Muslims being treated “rude and disrespectful” (E4, 30y).

The Arabic-speaking participants did not share experiences of language bias and had overwhelmingly positive experiences to share when asked about feelings of discrimination. One participant explained, “I don’t feel that [non-Muslims are treated better] no…to be honest I would like to thank the Americans in this regard…they respect you” (A6, 47y). Another participant agreed saying, “I have been here 17 years and I have never felt [discriminated against in healthcare]” (A2, 34y). All of these sentiments applied to their reproductive healthcare, as well as their healthcare experiences in general.

*II. Assumptions about Muslim identity.* Although most participants did not feel religiously discriminated against, some shared feeling stereotyped by clinicians. Participants explained how visible religious expressions, such as wearing the hijab, led to clinicians making assumptions about their preferences. “When you walk in and you’re covered…a male doctor will walk in, they do get a little ‘Are you okay with [me being here]?’…but not if it wasn’t a covered woman or a Muslim woman” (E2, 31y). Another participant shared “just because they see you covered up doesn’t mean you don’t speak their language. I think they have that mentality but it’s far from the truth” (E8, 27y). These assumptions may affect the care Muslim patients receive, as one participant mentioned “if they see I don’t have a wedding ring on and I’m Muslim, they’re like ‘Oh, so you’re not pregnant for sure…she’s a virgin. She doesn’t drink. I’m going to quickly go over this question’…whereas, people can be of varying levels of religiosity depending on whether they wear the headscarf or not” (E15, 24y).

#### Impact of Muslim Identity on Reproductive Health Preferences, Beliefs and Behaviors

*I. Preference for a female clinician tends to be specific to reproductive health*. Most participants preferred a female clinician for discussions about reproductive health and reproductive physical examinations. Participants explained that “it is difficult for Arab women and especially those who wear hijab to discuss [reproductive health] with male doctors” (A1, 29y). Another participant agreed: “I always prefer if it’s a woman if they are going to check private parts and stuff” (E5, 50y).

The Arabic-speaking participants were more fixed than the English-speaking participants about their preferences for reproductive health clinician gender. As one participant said, “I can’t expose my genital area to a male doctor. That’s impossible…that is not acceptable” (A8, 47y). Another participant indicated that in the past because of a male clinician “[I] did [refuse a gynecological exam] one time” (A5, 37y). Another Arabic-speaking participant explained how the preference was influenced by traditions more than just personal choice as, “some women don’t accept if the doctor is male because of their traditions. In Iraq, if we have an emergency, we accept to be examined by a male doctor but when it comes to labor or gynecological emergencies, some women can’t accept because their husband don’t accept or because their religion or families” (A1, 29y). However, participants overwhelmingly agreed that in an emergency “if there is only a male doctor, I would accept that. I have no choice” (A2, 34y). Other exceptions to the preference for a female clinician only appeared in the English interviews, as two participants mentioned that they actually preferred male clinicians because “they’re very gentle” (E4, 30y) and “women have a tendency to overthink” (E10, 54y). They did not provide further context into these beliefs.

When participants were asked to describe the best person to care for their reproductive health, almost all participants discussed character traits rather than gender. These included traits like “listen[ing] and understand[ing]” (E12, 47y) and “as long as they’re qualified and have a heart and can understand what I’m going through” (E10, 54y). Gender preference seemed to be related only to reproductive health, as most participants reported that “if I am having a gynecological exam, it should be a female doctor. But for any other problems, there is no difference between male or female doctors” (A3, 57y). Another participant concurred that “outside of the emergency [sic], if it is something related to OBGYN, I would prefer a female doctor only. But for any other health problems, it doesn’t matter if it is a male doctor” (A7, 27y).

*II. Preference for a non-Muslim/non-Arab clinician.* Multiple participants discussed a desire for a non-Muslim/non-Arab clinician while receiving reproductive healthcare, citing concerns about privacy. One participant explained that discussing her reproductive health with a Muslim or Arab clinician would cause “fear or worry [about] gossip in the community” (E15, 24y). Another participant agreed that “subconsciously…[Muslim or Arab clinicians] will judge you regardless of if they tell you they won’t” (E2, 31y).

Some participants shared beliefs that clinicians who did not come from their community were better. For example, one participant suggested that “people are so judgmental, and I’m Arabic, so trust me I know how they are…I love my race… but I’d rather have a [clinician be a] nice white lady or a nice black guy, Chinese, whatever…they’re good” (E6, 47y). Another participant concurred, “I think that Americans have more mercy than Arabs” (A8, 47y).

*III. Marital status impacts ideas about reproductive health.* For many participants, marital status was vitally important to reproductive health, because it provides an important context for the role intercourse plays in their religion, health, and healthcare. Many interviewees emphasized that “[intercourse] is [generally not permissible] in our religion…only with her husband” (A8, 47y). For some participants, women who “had sex and [weren’t] married…there’s no way in hell [they] could tell that to anybody” (E3, 33y), indicating that the subject of sexual activity is taboo and may not be discussed readily, if at all, by some Muslim women. Another participant explained this further, describing, “I broke my virginity and I wasn’t married. But I would still go talk to my gynecologist [who was] an old white guy…And I made sure that I didn’t know nobody that worked at that office” (E3, 33y). Similarly, marital status also influenced ideas about general reproductive health topics like menstruation since “Muslim girls don’t wear tampons if they’re not married yet” (E15, 24y). Another participant agreed, “you’re not supposed to do [pelvic exams] until basically you’re married” (E9, 20y).

*IV. Religious permissibility for contraception.* When participants were specifically asked how their religious beliefs impacted their choices around contraception, the responses were overwhelmingly positive and emphasized religious permissibility. As one participant explained, “No, [birth control] won’t be considered against God or religion because God knows how my health is. He knows that I can no longer tolerate another pregnancy…We all know ourselves, maybe we aren’t able to raise the children and to be responsible about them. These pills aren’t against Islam at all” (A2, 34). Another participant emphasized how these choices are personal since, “I rather not [use birth control] because I think [if a pregnancy is] meant to be, then it’s meant to be…[These feelings are] just personal. If birth control works for you then use it” (E6, 27). Not only did participants express religious permissibility, but one participant articulated religious necessity for the non-contraceptive effects of contraception. “When I was like 14, we went to the Islamic pilgrimage, Hajj. And to do that, I had to get on birth control so that I don’t get my period there and miss the opportunity to pray there” (E15, 24).

## DISCUSSION

In this study, we aimed to explore Muslim women’s reproductive healthcare experiences, preferences, beliefs, and behaviors. We were able to elucidate key themes from participants that can inform culturally sensitive care for this population. More specifically, this exploration highlighted the high proportion of Muslim representation in healthcare in the community we sampled, which helped mitigate discrimination and promoted inclusion. Previous studies in minority populations have highlighted the need for cultural representation and sensitivity as tools to better serve a community.^16^ Yet there were drawbacks to the insular nature of this community, such as the potential for loss of confidentiality that may lead to stigma around reproductive health behaviors. This influenced preferences for non-Muslim clinicians for some participants. Other studies have shown that minority communities receive better care from members of their own community, but that cultural concordance is not always possible.^16^^–^^19^ When not possible, environments should be intentional in hiring a diverse workforce to help increase the cultural knowledge of clinicians outside the minority population. All clinicians, including those who are religiously and culturally concordant, should explore and be sensitive to their patients’ concerns about privacy, especially for taboo subjects like reproductive and sexual health.

Interestingly, there were major differences that dichotomized the results between English- and Arabic-speaking participants, emphasizing the diversity of the Muslim population. The concerns about privacy and judgment were almost exclusively conveyed by the English-speaking participants. The English-speaking group was younger, more educated, and more likely to be native to the US compared to the Arabic-speaking group. In this way, the English-speaking participants may exhibit less traditional beliefs and behaviors than other subgroups in their community, such as older or immigrant women. The fact that members of the Arabic-speaking group were more fixed in their preference for a female clinician supports the idea that this group may be more traditional. Although religiosity was explicitly explored in our demographic survey, this is a term that is both subjective and relative, making it difficult to measure. This potentially explains the discrepancy between our hypothesis and the results finding that the English-speaking group reported more traditional religious beliefs.

Additionally, the Arabic-speaking participants overall did not endorse feelings of discrimination while receiving reproductive healthcare, despite the English-speaking participants reporting that language barriers played a role in their observation of discrimination against Arabic-speaking Muslim women. The Arabic-speaking group was exclusively born outside the US and likely had more experiences receiving reproductive healthcare overseas. Their positive reproductive healthcare experiences, contextualized in this US-based study setting, may be attributed to social desirability bias or ideas about superior healthcare in the US.^20^ Furthermore, since the English-speaking group predominantly identified as Lebanese, whereas the Arabic-speaking group were largely Yemeni, it is difficult to elucidate whether these differences were truly related to Muslim identity and religiosity or rather inherent ethnic dissimilarities. Although this study was focused on exploring the experiences of Muslim women, it is important to consider the challenges in capturing data that can be purely attributed to religious identity without being confounded by ethnic or cultural nuances.

The majority of participants desired a female clinician for reproductive healthcare in the ED unless it was an emergency situation, consistent with previous studies that have demonstrated this preference in almost all groups, regardless of race or religion.^21^ Yet many of the participants discussed certain personality traits, such as empathy and good listening skills, as the most important attributes when describing the best clinician for reproductive healthcare in the ED. This is an idea that has been established in previous studies, suggesting that it is not unique to Muslim patients to prioritize clinician qualities over gender for reproductive healthcare.^22^^,^^23^ Additionally, most participants agreed that they did not have a gender preference when receiving non-reproductive healthcare. Therefore, clinicians should avoid assumptions about gender preference in the Muslim patient population. This also emphasizes the need for gender diversity in healthcare when possible and creating policies to support patient clinician preferences for reproductive health when it can be feasibly accommodated.

Marital status was also found to dictate what was permissible regarding reproductive health. Participants discussed how sexual activity outside of marriage was largely considered taboo with major potential consequences within the Muslim community. This also influenced concerns about menstruation and pelvic exams compromising virginity. This is important knowledge for clinicians because they may be a patient’s only confidant on these subjects. It is also an area where clinicians can provide sexual health education and address misinformation with sensitivity to deeply held beliefs.

Lastly, this study helped elucidate Muslim women’s attitudes toward family planning, an area well known to be influenced by religious and cultural norms.^24^^–^^26^ Participants conveyed beliefs about the religious permissibility of contraception, as well as in some cases, religious necessity. This is in line with a recent self-reported survey that examined American Muslim’s contraception utilization patterns, which demonstrated that Muslim respondents reported higher contraception use than the national proportion.^27^ Participants also emphasized that choices about contraception were personal and should not be influenced by others’ beliefs. These are important takeaways because they demonstrate that family planning counseling should be tailored to an individual’s motivations and goals, rather than based on assumptions about cultural or religious belief.^28^

Previous literature that has focused on cultural competency in providing medical care to the Muslim population has largely included generalizations about the Muslim population, such as preference for a female clinician or assumptions about sexual activity before marriage.^29^^–^^31^ This study focused on individual experiences that at times contradicted these generalizations. Our finding aligns with the cultural empowerment model of cultural competence that emphasizes the dynamic nature of cultural competency.^15^ More specifically, “because of the specific nature of each patient-clinician interaction within its particular social and political environment, culturally competent behavior in one context may be culturally incompetent in another.”^15^ This provides a framework for providing care to the Muslim population who exhibit a large range of diversity—such as race, ethnicity, and language—that may heavily influence the way one practices their religion.

## LIMITATIONS

This study had several limitations. We enrolled English- and Arabic-speaking patients from a single ED within a predominantly Middle Eastern community; therefore, the results may not be generalized to all Muslims. However, the qualitative approach was designed to be exploratory and generate hypotheses and future research questions that may be evaluated for generalizability in the future. The demographic survey and interviews in Arabic may have been affected by participant comprehension and literacy or translation nuances that changed the meaning of concepts, which is a limitation of the study. Additionally, the demographics of the interviewers may have affected the sentiments our study participants felt comfortable sharing due to social desirability bias. More specifically, AN was younger and not visibly Muslim, so participants may have spoken more freely about stigmatizing topics with her than with MS, a lifelong member of the local Muslim community who is a nurse at the study site.

## CONCLUSION

Our findings contribute to the growing body of literature focusing on cultural sensitivity in treating the Muslim population. We found that cultural representation and sensitivity among ED staff mitigated discrimination and promoted inclusion for Muslim ED patients. However, assumptions about Muslim identity also impacted the participants’ healthcare. Most participants endorsed a preference for a female clinician for their reproductive healthcare in general, but not necessarily for other areas of medicine. Clinician cultural concordance was not always preferred by participants in this ED study due to fears about the loss of confidentiality. Marital status impacted beliefs about reproductive and sexual health in the context of Muslim identity. Overall, family planning was acceptable and encouraged in this patient population.

This study is unique because it emphasizes patient preferences and focuses on female Muslims’ reproductive health preferences, an area of clinical importance that has not been thoroughly explored. Ultimately, our findings underscore the need for future work to capture a more diverse perspective of Muslim women and better elucidate the reproductive health preferences and needs that are unique to this population.
